# Paired RNA Radiocarbon and Sequencing Analyses Indicate the Importance of Autotrophy in a Shallow Alluvial Aquifer

**DOI:** 10.1038/s41598-019-46663-1

**Published:** 2019-07-17

**Authors:** Brian J. Mailloux, Carol Kim, Tess Kichuk, Khue Nguyen, Chandler Precht, Shi Wang, Talia N. M. Jewell, Ulas Karaoz, Eoin L. Brodie, Kenneth H. Williams, Harry R. Beller, Bruce A. Buchholz

**Affiliations:** 1 Environmental Science Department, Barnard College, NY, NY 10027 New York, USA; 2 Earth and Environmental Sciences, Lawrence Berkeley National Laboratory, Berkeley, CA 94720 USA; 3 Center for Accelerator Mass Spectrometry, Lawrence Livermore National Laboratory, Livermore, CA 94551-9900 USA

**Keywords:** Microbial ecology, Molecular biology, Water microbiology, Biogeochemistry

## Abstract

Determining the carbon sources for active microbial populations in the subsurface is a challenging but highly informative component of subsurface microbial ecology. This work developed a method to provide ecological insights into groundwater microbial communities by characterizing community RNA through its radiocarbon and ribosomal RNA (rRNA) signatures. RNA was chosen as the biomolecule of interest because rRNA constitutes the majority of RNA in prokaryotes, represents recently active organisms, and yields detailed taxonomic information. The method was applied to a groundwater filter collected from a shallow alluvial aquifer in Colorado. RNA was extracted, radiometrically dated, and the 16S rRNA was analyzed by RNA-Seq. The RNA had a radiocarbon signature (Δ^14^C) of −193.4 ± 5.6‰. Comparison of the RNA radiocarbon signature to those of potential carbon pools in the aquifer indicated that at least 51% of the RNA was derived from autotrophy, in close agreement with the RNA-Seq data, which documented the prevalence of autotrophic taxa, such as *Thiobacillus* and *Gallionellaceae*. Overall, this hybrid method for RNA analysis provided cultivation-independent information on the *in-situ* carbon sources of active subsurface microbes and reinforced the importance of autotrophy and the preferential utilization of dissolved over sedimentary organic matter in alluvial aquifers.

## Introduction

Shallow aquifers are often characterized as oligotrophic environments in which a heterotrophic microbial lifestyle is supported by surface-derived allochthonous organic matter^1^, although recent studies have demonstrated the importance of chemolithoautotrophy in some aquifer environments^2–4^. Determining the source of allochthonous and/or autochthonous carbon utilized by microbes can be difficult in aquifer systems. Potential carbon sources include dissolved organic carbon (DOC) transported from the surface^5^, dissolved inorganic carbon (DIC) from recharge, respiration, or mineral dissolution^6^, carbon associated with mineral grains (sediment organic carbon and adsorbed organic carbon)^7,8^, and carbon-rich material deposited with sediment (e.g., buried plant material or peat layers)^9–12^. Understanding these sources is critical for constraining the biogeochemical reactions that can control water quality and biogeochemical feedbacks to the atmosphere^13–15^.

RNA profiling with next-generation sequencing technologies (RNA-Seq) and radiocarbon analysis of RNA offer insights to constrain carbon utilization by microbes in a shallow aquifer, but to our knowledge these techniques have not yet been used in combination. DNA- or RNA-sequence-based techniques have been used to infer heterotrophic vs. autotrophic lifestyles. Examples include targeting genes for diagnostic taxa (e.g., use of 16S rRNA gene-based primers to target lithoautotrophic, Fe(II)-oxidizing *Gallionella*)^16^ or targeting genes/transcripts for diagnostic metabolic pathways, for example, for RubisCO of the Calvin-Benson-Bassham cycle^3,17,18^ or for the reductive tricarboxylic acid cycle^6^. In addition to such targeted (e.g., PCR-based) studies, metagenomic and metatranscriptomic studies have also revealed information about heterotrophic vs. autotrophic lifestyles in the subsurface. For example, genome reconstruction based upon metagenomic data from aquifer sediment has strongly indicated an autotrophic lifestyle for prominent but uncultivated subsurface microorganisms^19^. Metatranscriptomic (RNA-Seq) and metagenomic analysis of an alluvial aquifer near Rifle, CO, highlighted the unexpected abundance and activity of a small group of autotrophic taxa both before and during injection of nitrate into a suboxic aquifer^2^. Still, comparatively few studies have sequenced RNA from the subsurface to better understand the importance of autotrophy vs. heterotrophy among uncultivated subsurface microbes.

Radiocarbon analysis is a sequence-independent method that has been used to determine carbon sources in surface waters and in the subsurface, based on the study of targeted biomolecules, such as phospholipid fatty acids (PLFA) and DNA^7,20–23^. Such radiocarbon techniques are most powerful when different carbon sources (e.g., organic and inorganic carbon) have distinct radiocarbon signatures. PLFA are components of the cell walls of active microbial populations and can provide information on bulk microbial populations in addition to microbial functional groups. PLFA are typically analyzed in sediment samples^24,25^ but have also been analyzed in aqueous samples^26,27^. Radiocarbon analysis of DNA represents carbon utilized for cell division but has the potential to represent both active and non-viable cells^28^ and also potentially long-lived ‘relic-DNA’^29^. RNA is conventionally thought to offer advantages over DNA for representing active, or recently active, microbes, although there are caveats for equating rRNA with actively growing or metabolizing cells^30^. In aquifer systems, radiocarbon analysis has indicated the importance of allochthonous carbon, such as petroleum products in contaminated aquifers^31^, and advected DOC in alluvial aquifers with geogenic arsenic^21,32^. In the deep subsurface, radiocarbon analysis of PLFA has been able to identify autotrophy^23,27^. Stable isotope analysis, but not radiocarbon analysis, has been performed on environmental RNA samples^33,34^.

The goal of this work was to develop and test a cultivation-independent method that analyzes both the radiocarbon and rRNA signature of *unamplified* RNA from the same environmental sample, and that collectively characterizes community carbon utilization under *in situ* conditions. The method was applied to a filtered groundwater sample from a geochemically reduced zone of a shallow alluvial aquifer near Rifle, CO, where multiple potential carbon sources co-occur. The radiocarbon and RNA-Seq results, while addressing diverse sample properties, indicated a strikingly similar conclusion about the quantitative role of autotrophy in the subsurface microbial community.

## Results and Discussion

### Radiocarbon Method Validation Using *Escherichia coli* Grown with Characterized Carbon Sources

Growth media that contained carbon with end-member radiocarbon signatures [acetate, lysogeny broth (LB), and dextrose] were utilized to validate the RNA radiocarbon purification method. Commercially available acetate is derived mostly from petroleum and thus possesses a highly negative radiocarbon signature (Δ^14^C) and a comparatively old C age. In practice, a sample with little to no radiocarbon has a Δ^14^C value approaching −1000‰ and contains carbon more than 50,000 years old. In contrast, LB and dextrose are derived from recent photosynthetically derived material and have a contemporary signature with a positive Δ^14^C value and a modern age (Table 1). Above-ground testing of nuclear weapons before the adoption of the Limited Test Ban Treaty of 1963 produced a bomb pulse of ^14^C in the atmosphere resulting in radiocarbon values with a fraction modern greater than 1 and a positive Δ^14^C value^35^. The use of the different growth media in the laboratory offered a controlled approach to validate the extraction and radiocarbon analysis procedure.

**Table 1:** Summary of Radiocarbon Results.

Medium	Surfac-tant	P:C pH^a^	P:C Tubes^b^	LiCl^c^	CsCl^d^	Fraction Modern	Δ^14^C (‰)	^14^C age (yrs)	260/280^e^	260/230^e^	Percent DNA (%)^f^	Amount RNA (μg)^g^	CAMS^h^
100% EtOH		Pure Medium, No Prep				1.0491 ± 0.0037	40.8 ± 3.7	>Modern					173559
100% EtOH & DEPC		Pure Medium, No Prep				1.0504 ± 0.0037	42 ± 3.7	>Modern					173560
CTAB in water		Pure Medium, No Prep				0.0048 ± 0.0001	−995.2 ± 0.1	42900 ± 170					174198
LB medium		Pure Medium, No Prep				1.0675 ± 0.0039	59.9 ± 3.7	>Modern					143162
Acetate		Pure Medium, No Prep				0.1414 ± 0.0008	−860 ± 1	15,715 ± 45					149428
Dextrose		Pure Medium, No Prep				1.0633 ± 0.0035	55.6 ± 3.5	>Modern					149098
*E. coli*		Pure Medium, No Prep				1.0302 ± 0.0039	24.8 ± 3.9	>Modern					174199
*E. coli* washed^i^		Pure Cells, No Prep				1.0321 ± 0.0043	26.6 ± 4.3	>Modern					174200
LB	SDS	7.9	small	no	no	1.0094 ± 0.007	1.6 ± 7.0	>Modern	na	na	na	na	167573
LB	SDS	7.9	small	yes	no	1.0021 ± 0.0067	−5.7 ± 6.7	>Modern	na	na	na	na	167574
LB	SDS	7.9	small	yes	yes	0.9687 ± 0.0132	−38.7 ± 13.2	260 ± 110	na	na	na	na	167575
LB	SDS	7.9	small	yes	yes	0.9639 ± 0.0255	−43.5 ± 25.5	300 ± 220	na	na	na	na	167676
LB	CTAB	6.6	small	no	no	0.9711 ± 0.0038	−36.7 ± 3.8	235 ± 35	2.08	2.1	10.6	197	176279
LB	CTAB	6.6	small	no	yes	0.9259 ± 0.0061	−81.3 ± 6.1	620 ± 60	1.99	1.99	<1%	na	172417
LB	CTAB	6.6	small	no	yes	0.9351 ± 0.0044	−72.2 ± 4.4	540 ± 40	2.06	2.18	<1.3%	na	172418
LB	CTAB	6.6	small	no	yes	0.8836 ± 0.0041	−123.5 ± 4.1	995 ± 40	1.88	2.17	5	na	173313
LB	CTAB	6.6	small	no	yes	0.9297 ± 0.004	−77.7 ± 4	585 ± 35	1.92	2.23	<1%	na	173315
LB	CTAB	6.6	small	no	yes	0.9189 ± 0.0089	−88.4 ± 8.9	680 ± 80	1.98	2.34	2.7	60	175675
LB	CTAB	6.6	large	no	no	0.9862 ± 0.0038	−21.7 ± 3.8	110 ± 35	2.12	2.07	10.0	283	176278
LB	CTAB	7.9	large	no	no	0.9854 ± 0.0047	−22.6 ± 4.7	120 ± 40	2.05	1.95	6.8	164	176274
LB	CTAB	7.9	large	yes	no	1.0000 ± 0.0043	−8 ± 4.3	Modern	2.07	2.05	0.7	127	176277
LB	CTAB	7.9	large	yes	no	1.0056 ± 0.006	−2.5 ± 6	Modern	2.07	2	0	62	176275
LB	CTAB	7.9	small	no	no	1.0236 ± 0.0069	15.7 ± 6.9	>Modern	2.03	2.24	na	83	167569
LB	CTAB	7.9	small	yes	no	1.0277 ± 0.0054	19.8 ± 5.4	>Modern	2.05	2.28	4.7	143	167570
LB	CTAB	7.9	small	yes	no	1.0032 ± 0.007	−4.7 ± 7	Modern	2.01	1.72	0.5	na	169942
LB	CTAB	7.9	small	yes	no	1.0125 ± 0.0164	4.3 ± 16.4	>Modern	2.03	2.28	1.6	21	176276
LB	CTAB	7.9	small	yes	no	1.0145 ± 0.0044	6.3 ± 4.4	>Modern	na	na	na	340	176271
LB	CTAB	7.9	small	yes	yes	1.012 ± 0.0111	4.2 ± 11.1	>Modern	2.00	2.4	3.8	49	167571
LB	CTAB	7.9	small	yes	yes	1.0235 ± 0.0043	15.6 ± 4.3	>Modern	2.09	2.1	na	203	167572
Acetate	CTAB	7.9	small	yes	no	0.1629 ± 0.0053	−838.4 ± 5.3	14580 ± 270	2.03	2.17	0.6	na	169940
Dextrose	CTAB	7.9	small	yes	no	1.046 ± 0.0042	37.8 ± 4.2	>Modern	2.1	2.26	1.1	na	169941
LQ107-RNA −50 μgC	CTAB	7.9	small	yes	no	0.8129 ± 0.0056	−193.4 ± 5.6	1660 ± 60	1.95	2.13	na	na	169943

Results are shown for growth media, chemicals used for RNA extraction, pure cells, and extracted RNA with the steps for each extraction indicated. ^a^The pH of Phenol:Chloroform. ^b^Type of Phenol:Chloroform tube utilized. ^c^Utilization of a LiCl precipitation step. ^d^Utilization of CsCl ultracentrifugation. ^e^The spectrophotometric ratio of the RNA to determine purity measured on a NanoDrop. Values near or above 2.0 indicate nucleic acids free of contaminants. ^f^The percent DNA determined fluorescently with a Qubit Fluorometer. ^g^The amount of RNA was estimated from the NanoDrop concentrations. ^h^The CAMS accession number. ^i^*E. coli* was washed and/or resuspended in DNA-grade sterile water.

Twenty-four RNA samples extracted from *E. coli* grown with the different media were analyzed for their radiocarbon signatures to develop the current method (Table 1), which was adapted from previously described methods for isolating DNA^21,35^. Whole *E. coli* cells grown with LB and suspended in sterile water before the extraction procedure were less positive in Δ^14^C than the medium, but still modern (Table 1). Initial testing indicated that presence of SDS (sodium dodecyl sulfate) as a surfactant could not reproduce the modern signatures (three samples had a negative Δ^14^C value), so CTAB (hexadecyltrimethyl ammonium bromide) was used instead (Table 1). In addition, RNA purification columns were avoided, as they can contaminate samples with variable amounts of petroleum-derived carbon^36^. *E. coli* grown with acetate had an RNA radiocarbon signature close to that of the pure acetate medium (Table 1). However, subtle changes to the procedure to optimize RNA recoveries had a substantial impact on the radiocarbon signature of *E. coli* controls grown with LB. Seven samples extracted using water-saturated phenol (pH 6.6) all produced non-modern, more negative RNA signatures than the cells (Table 1). This was independent of the purification steps, which included multiple types of phase-lock gel tubes, ethanol washes, and CsCl centrifugation. None of these purification steps could remove the petroleum-derived contamination from the RNA. Water-saturated phenol is generally recommended for RNA extractions, as it increases yields of RNA while removing DNA^37^; however, in this case, residual C from the phenol treatment could not be fully removed, skewing the radiocarbon signature of the RNA.

Ten samples were extracted using Tris-saturated phenol (pH 7.9) (Table 1). When using the large phase-lock gel tubes with LiCl, the Δ^14^C values of the three samples were all negative, although two were considered modern. Seven samples using the small phase-lock gel tube all had Δ^14^C signatures that were modern, with only one negative value (Table 1). The results were highly reproducible with a mean and standard deviation of 8.7 ± 8.6‰, with the standard deviation close to the average analytical error of 7.9‰. The use of CsCl centrifugation did not change the Δ^14^C signature, but this step was omitted because of the resulting loss of RNA. In addition, after the LiCl step, the spectrometric 260/230 nm and 260/280 nm ratios indicated that the RNA was largely free of contaminants, with almost all values greater than 2.0 (Table 1). The amount of DNA as determined by fluorescence was low, <5%, in all samples (Table 1). The final recommended procedure used phenol at pH 7.9, the small phase-lock gel tubes, and LiCl.

The final method still produced a Δ^14^C value that was more negative than the LB and the washed cells (Table 1). The average Δ^14^C value for the four RNA samples using the final method was 6.4‰, compared to the average *E. coli* cells of 25.7‰, a difference of 19.3‰. Trials included *E. coli* cells grown with dextrose in M9 minimal medium to simplify conditions relative to LB (i.e., growth medium containing a single carbon source). For this medium, the Δ^14^C value of *E. coli* RNA was 37.8 ± 4.2‰, compared to the dextrose medium value of 55.6 ± 3.5‰, a difference of 17.8‰. Whole *E. coli* cells grown with dextrose were not analyzed, but given the single carbon source, we assume that the signature of *E. coli* cells would have been similar to the RNA and dextrose. It is not clear if the consistent 17–20‰ difference between the radiocarbon signatures of RNA vs. the growth medium (for LB or dextrose) was due to petroleum-derived contaminants in the RNA or isotopic fractionation during growth. For example, there could be relic carbon from the extraction buffers or carbon leaching from plastic or the Milli-Q system that was not removed. An extraction without cells did not have enough carbon for radiometric analysis and could not be used to trace exogenous carbon sources. Regardless, the radiocarbon signatures of the RNA and the medium/cells were consistent and comparable, and indicated that the method could be used to assess a range of carbon sources in the environment.

### Rifle aquifer

#### Radiocarbon data

Monitoring well LQ107 is located within a so-called “naturally reduced zone” of a shallow alluvial aquifer near Rifle, CO^38^. The well was sampled to determine if radiocarbon and RNA-Seq analyses could be combined to better understand carbon sources for microbes in aquifer systems. Approximately 23,520 L of groundwater were filtered (0.2-μm) and the RNA was extracted from the filter for radiocarbon and sequence analysis.

**Figure 1:**
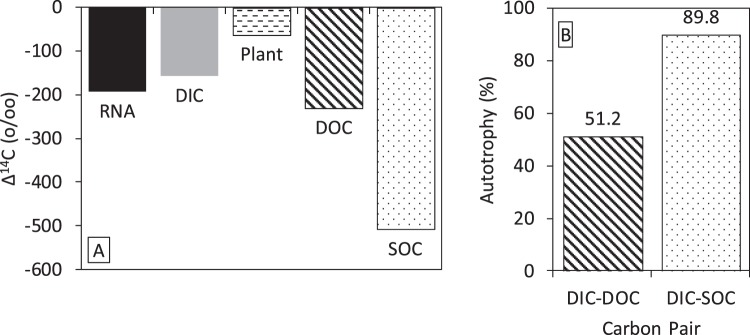
Summary of radiocarbon data from Well LQ107, a shallow alluvial aquifer near Rifle, CO. (**A**) Δ^14^C values of parameters measured from the well and aquifer. (**B**) Estimates of the amount of RNA from autotrophy from the DIC-DOC and DIC-SOC pairs in panel A. The plant carbon cannot be paired with DIC, as the plant radiocarbon is more positive than both DIC and sample RNA. The sediment and plant data are from Janot *et al*.^12^ and all the data are presented in the supplementary information. The RNA, DIC, and DOC are based on single samples whereas the plant (*n* = 8) and SOC (*n* = 20) are based on multiple samples.

The radiocarbon data from well LQ107 are summarized in Fig. 1. The Δ^14^C value of the RNA was −193.4 ± 5.6‰. The Δ^14^C values of the DIC and DOC were −157.7 ± 1.7‰ and −231.0 ± 1.7‰, respectively. In the aquifer, the average Δ^14^C value of the sediment organic carbon (SOC) and plant material were −504.4 ± 173.0‰ (*n* = 20) and −55.1 ± 61.8‰ (*n* = 8), respectively^12^. The range in radiocarbon values for potential carbon sources is much larger than the variability in radiocarbon signatures associated with the *E. coli* controls, indicating the method can be utilized to determine *in situ* carbon sources. Assuming that the RNA represents carbon from a linear combination of autotrophy and heterotrophy, the percent of RNA carbon from DIC was calculated with a DOC or SOC end-member. The DIC-DOC pair indicated 51.2% autotrophy and the DIC-SOC pair indicated 89.7% autotrophy. This assumes no contribution from buried plant material, as this plant material could not have been the primary source of carbon for the RNA, given the radiocarbon signatures (Fig. 1). However, the exact source of the DOC is not known and could be a mixture of carbon leaching from the soil horizon and buried plant material, along with the older sedimentary organic carbon. In this analysis, we used end-member radiocarbon signatures as the most parsimonious approach, but acknowledge that DOC is not a homogeneous compound but rather a heterogeneous mixture from multiple sources, each with potentially different bioavailability and/or radiocarbon signatures^39^. Nonetheless, as described below, the rRNA sequencing analysis supports the conclusions of the linear end-member mixing approach used for radiocarbon.

#### Community rRNA sequencing results

**Figure 2:**
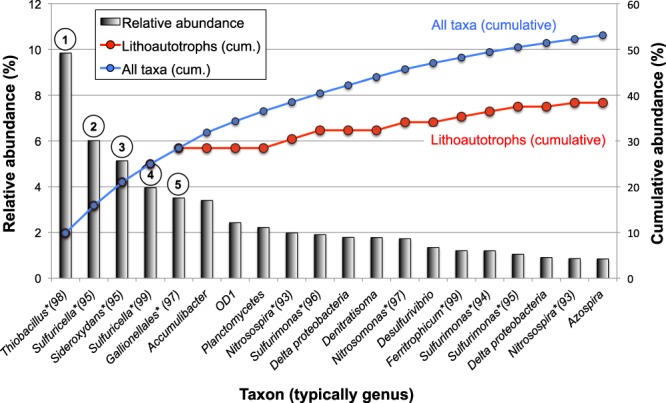
RNA-Seq results showing the relative abundance of the top 20 reconstructed sequences in the Rifle aquifer RNA sample, along with cumulative abundance of all sequences (blue) and lithoautotrophic sequences (red). Closest matching taxa based on 16S rRNA analysis are indicated on the *x*-axis (asterisks indicate lithoautotrophic taxa and numbers in parenthesis indicate % sequence identity to best 16S rRNA match). See text for discussion of assignment of heterotrophic versus autotrophic taxa. Phylogenetic relationships of the five most abundant taxa (indicated with circled numbers) are shown in Fig. 3.

**Figure 3:**
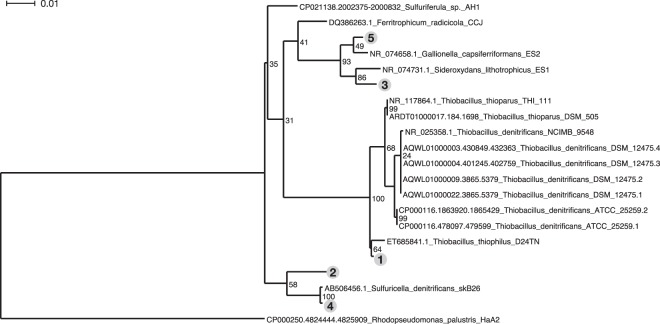
Phylogenetic relationships of the five most abundant taxa to the most closely related isolates, which have been experimentally confirmed as lithoautrophic bacteria (see text and Fig. 2). Numbers preceding isolate species names are GenBank accession numbers for their 16S rRNA gene sequences, and circled numbers correspond to taxa indicated in Fig. 2. *Rhodopseudomonas palustris* HaA2 is used to outgroup root the tree. Node confidences based on bootstrapped trees are noted next to the nodes. The scale bar represents percent sequence difference.

Sequence analysis of an aliquot of the RNA sample from Well LQ107 revealed the community composition of putatively active microbes at the time of sampling (Supplementary Data Files 1 and 2). A striking aspect of the RNA-Seq analysis was the predominance of lithoautotrophic bacteria (i.e., bacteria that use CO_2_ as a carbon source and inorganic compounds as electron donors) among the most abundant taxa (Fig. 2). The five most abundant taxa were all closely related to lithoautotrophic members of the β-proteobacteria that use reduced S compounds (such as H_2_S) and/or Fe(II) as electron donors, including the following (Figs 2 and 3): *Thiobacillus* (e.g.^40–42^), *Sideroxydans* and/or other *Gallionellaceae* family members (e.g.^43^), and *Sulfuricella* (e.g.^44,45^). The close phylogenetic relationships among the five most abundant taxa and experimentally characterized lithoautotrophic, β-proteobacterial isolates are depicted in Fig. 3. Other lithoautotrophic, S-oxidizing bacteria occurring among the 20 most abundant taxa included *Sulfurimonas* spp., which belong to the ε-proteobacteria (e.g.^46^) (Fig. 2). *Nitrosospira* and *Nitrosomonas* spp., which can use ammonia as an electron donor, were also present among the top 20 taxa (Fig. 2). Overall, among the 20 most abundant taxa in the sample, bacteria closely related to known lithoautrophic bacteria (with 96% average sequence identity) constituted ca. 72% of the population (Fig. 2). If this analysis is expanded to all taxa that constituted at least 0.1% of the community (139 taxa and 80.7% of all taxa detected; Supplementary Data File 1), then bacteria closely related to known lithoautrophs constituted ca. 52.5% of the population.

Thus, RNA-Seq analysis provided strong evidence of lithoautotrophic bacterial activity in the Rifle aquifer. Notably, these results are very consistent with a recent study of the Rifle aquifer^2^, in which Fe(II)- and S-oxidizing lithoautotrophic bacterial strains belonging to *Sulfurimonas* and the *Gallionellaceae* were found to be highly active, collectively accounting for ca. 80% of the metatranscriptome (community gene expression). Further, a different Rifle aquifer study indicated the predominance of another S-oxidizing lithoautotrophic bacterium, *Candidatus* Sulfuricurvum sp. RIFRC-1, that constituted ca. 47% of the aquifer sediment community based upon metagenomic data^19^.

#### Combining radiocarbon and RNA-Seq data

Radiocarbon and RNA-Seq analysis of RNA are both resource- and time-intensive methods that can currently only be performed on a small number of well-constrained samples, but together can offer novel insights. Targeting RNA in environmental samples makes processing more difficult than DNA analyses, but ensures a focus on predominantly active microbes. While PLFA analysis also targets active microbes, it provides much less detailed taxonomic information than rRNA sequencing. In this study, two methods that target RNA but independently provide estimates of carbon sources through either a geochemical measurement or a taxonomic assessment indicated the important role of autotrophy in a subsurface microbial community. This mirrors recent findings at Rifle and other sites^2,6,47^.

The data also provided information on the probable source of the organic matter used by heterotrophs. In order for the radiocarbon-based estimates of autotrophy (ca. 50 to 90%; Fig. 1) to match the amplification-free, 16S rRNA-based estimates (ca. 50%) (Fig. 2), heterotrophs would need to have used the end-member DOC as their carbon source, and the end-member SOC is probably not a major contributor to microbial respiration or biomass. This is consistent with other alluvial aquifers in which recent, advected carbon sources were preferentially utilized over sediment-associated carbon^21,32^. It is not clear why SOC is not utilized by the microbes in alluvial aquifers, but multiple ecosystem properties might account for this stabilization^39^, even though sediment organic matter could be bioavailable once it is released from the sediment^48^. In this shallow alluvial aquifer, it appears that heterotrophic redox reactions are supported by DOC, while reduced inorganic compounds mix with oxidants delivered to the aquifer during seasonal hydrologic fluctuations^49^ to support a thriving and diverse chemolithoautotrophic community that accounts for approximately half of the active, planktonic, microbial biomass. The combination of next-generation sequencing of unamplified 16 S rRNA with radiocarbon analysis of RNA could be a powerful, cultivation-independent method for understanding subsurface carbon dynamics under *in situ* conditions.

## Methods

### Site overview

**Figure 4:**
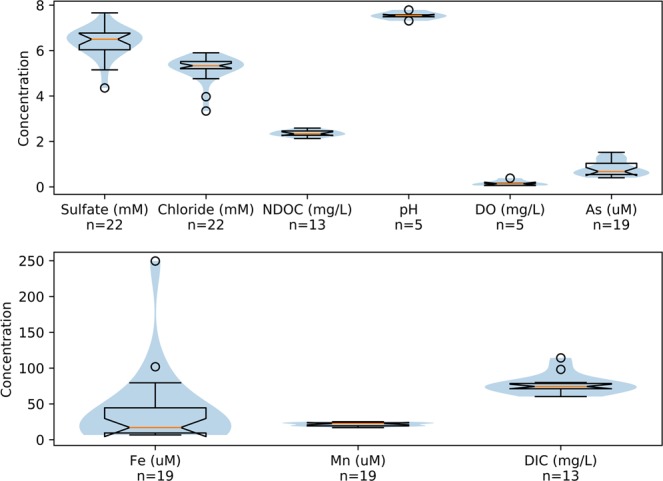
Summary of groundwater chemistry at Well LQ107 over a ca. 2-year period preceding sampling for the current study. The blue shading represents the distribution of the samples. The orange line represents the median, the notch in the box represents the 95% confidence level for the median, the edge of the box is the quartile range, the whiskers are the total data range, and the circles are outliers.

Groundwater samples were collected in August, 2011 from monitoring well LQ107, located in a shallow alluvial aquifer near Rifle, Colorado. The well is 6.1 meters deep and screened over the bottommost 3 meters. The water table varies from approximately 1.8 to 4.1 meters below the land surface, depending on river stage in the Colorado River, which adjoins the site to the south. An extensive overview of the site was presented by Williams *et al*.^50^. The well is located in the naturally reduced zone, as indicated by redox-sensitive elements (Fig. 4) with lithological and mineralogical properties analogous to those described by Campbell *et al*.^38^. Dissolved Fe and Mn were at elevated concentrations, along with arsenic. Sulfate is present and relatively stable over time. Microbially mediated Fe(III) and sulfate reduction at the site have contributed to the presence of reduced Fe- and S-containing compounds in the aquifer, including Fe-sulfide minerals, such as FeS and FeS_2_. DIC and DOC concentrations are relatively constant over time. The alluvial Rifle aquifer is underlain by the impermeable Wasatch Formation at a depth of 5.5 meters at well LQ107.

### Groundwater filtration

Groundwater samples were collected from monitoring well LQ107 in August 2011 using a peristaltic pump; groundwater was pumped through a 0.2-μm nylon 10-inch filter cartridge (Filtersource, Hamburg, NY) for 4 days at 3.8 liters per min for a total volume of 23,520 L. The high flow rate through the filter maintained the temperature of the filtered residuum at an aquifer temperature of ca. 13 °C over the sampling duration. The filter was kept in the dark during filtering to limit microbial activity, and then immediately frozen at −20 °C and shipped frozen to Barnard College, where it was stored at −80 °C until processing.

### RNA extraction and purification

The complete RNA extraction and purification procedure is presented in the SI. An abbreviated version that highlights less conventional portions is presented here. The frozen filter was cut into ~1-inch rings with a sterilized electric saw, then cut into small sections and the outer-shiny layer was placed in a 50-mL tube. The RNA extraction buffer was 2% CTAB, 1.4M NaCl, 20 mM EDTA (pH 8), and 100 mM Tris-Cl (pH 8), with 64 mg lysozyme and 16 mg proteinase-K added along with 1.7 g of 3-mm sterile beads. The mixture was vortexed, incubated for 30 min at 55 °C, decanted, and washed a second time with 1x Tris-EDTA (TE). The extract was purified using phenol:chloroform in two rounds with Phase Lock Gel (PLG) tubes (a.k.a. MaXtract High Density, Qiagen, Germantown, MD), which were made with a silicone lubricant^51^. The first round used 50-mL PLG tubes and the second round used either 50-mL or 1.5-mL PLG tubes (this was an important variable, as discussed earlier). In addition, the phenol was tested as both water-saturated (pH 6.6) and Tris-saturated (pH 7.9), and again, this was an important variable. The extracts were precipitated with isopropanol, washed with 70% ethanol, and resuspended in DNA-grade sterile water. The RNA was further purified with LiCl and/or CsCl ultracentrifugation (see SI). DNase treatment was not utilized at this point to avoid adding any excess carbon to the sample that could impact radiocarbon dating; however, DNase was used prior to RNA-Seq analysis, as described below. The purity and amounts of RNA were assessed with a NanoDrop Spectrophotometer (Thermo Fisher Scientific, Waltham, MA) and verified with a Qubit Fluorometer (Thermo Fisher) using both RNA and DNA kits. RNA was stored in DNA-grade sterile water at −80 °C until RNA-Seq or radiocarbon analysis.

#### RNA controls

Two end-member cases were used to test the RNA extraction procedure*. E. coli* (strain ATCC 700891, ATCC, Manassas, VA) was grown with either sodium acetate (Fisher Scientific, Pittsburgh, PA) or dextrose (Fisher) with M9 medium^52^ or lysogeny broth (LB) (Fisher). Acetate is mostly petroleum-derived and is an older end-member, whereas LB contains yeast extract as a carbon source and provides a modern radiocarbon end-member along with dextrose. Cells were grown and diluted multiple times (to dilute out previous carbon signatures), pelleted, resuspended in 5 mL of DNA-grade sterile water, and stored at −80 °C until 1 mL was used for extractions.

#### Radiocarbon analysis

Purified RNA samples suspended in water and process controls were shipped to Lawrence Livermore National Laboratory (LLNL) for accelerator mass spectrometry (AMS) sample processing and analyses through the natural carbon preparation laboratory. RNA storage is not critical for radiocarbon analysis, as storage at room temperature results in RNA degradation, but not mineralization. Samples were transferred to quartz combustion tubes and lyophilized to dryness. Isotopic standards NIST SM 4990C Oxalic Acid II and IAEA C-6 sucrose were dissolved in water to produce secondary standards with similar masses as the RNA and lyophilized with the samples. After removal from the lyophilizer, all dry samples, controls, and standards received excess CuO and were promptly evacuated and sealed with a H_2_/O_2_ torch. The sealed tubes were placed in a furnace set at 900 °C for 3.5 h to oxidize all carbon to CO_2_. The evolved CO_2_ was purified, trapped, and reduced to graphite using standard techniques^53,54^. Graphite samples were measured at the Center for AMS at LLNL using the HVEE FN AMS system operating similarly to our previous analyses of DNA^21^. Most samples were too small to obtain CO_2_ splits for δ^13^C analyses. Based on a few large sample splits, a fractionation correction of δ^13^C = −20 ± 2‰ was used for RNA samples. The measurement error was determined for each sample and ranged between ±4‰ and 11‰ (1SD) for samples with greater than 25 μg carbon. All data was reported as decay-corrected ∆^14^C^55^ and post-bomb F^14^C^56^.

#### Metatranscriptome analysis

RNA purification, RNA-Seq library construction, and sequencing: Samples for molecular analysis were kept at −80 °C and shipped on dry ice to Lawrence Berkeley National Laboratory. Total isolated RNA was further purified using the Qiagen RNeasy cleanup kit following the manufacturer’s instructions with a modification for DNase digestion: after the first wash of the RNeasy mini spin column with 250 μL buffer RW1, 80 μL of DNase I incubation mix (10 μL DNase I stock and 70 μL buffer RDD) was directly loaded onto the column and incubated at room temperature for 15 min. The purified sample was analyzed for quality with a Bioanalyzer 2100, using RNA 6000 Pico Chips (Agilent Technologies, Wilmington, USA) and was quantified with a Qubit fluorometer, using a Broad Range RNA kit (Agilent Technologies). The cDNA library was prepared with 60 ng total RNA and was constructed as described by Jewell, *et al*.^2^. Briefly, we used a TruSeq RNA Sample Preparation Kit v1 (Illumina), with a single modification: the protocol began with the “Elute, Prime, and Fragment” step by adding 13 μL of the “Elute, Prime, and Fragment Mix” to 5 μL of purified RNA. The cDNA library concentration was assessed using the KAPA SYBR FAST qPCR Kit following the manufacturer’s instructions (KapaBiosystems, Boston, USA). Quality was assessed with a Bioanalyzer using a DNA 1000 chip (Agilent Technologies). The sample was sequenced by the Vincent J. Coates Genomics Sequencing Laboratory at U.C. Berkeley on an Illumina HiSeq4000 with paired-end, 100-bp reads.

Ribosomal 16S rRNA sequence assembly: Near-full-length ribosomal 16S rRNA sequences were reconstructed from total RNA reads using a large database of candidate rRNA genes with a templated assembly approach (EMIRGE^57^). EMIRGE reference databases were constructed using the SILVA SSU SEED database (release 123)^58^ and 16S rRNA sequences from metagenome-assembled genomes^59^, resulting in a final candidate database of 13,386 sequences with taxonomic labels. EMIRGE was parameterized to run in single-end mode to iterate 40 times, to merge 97% identical sequences, and to accept the reference sequences that were at least 30% covered lengthwise. 16S rRNA gene relative abundances were estimated based on normalized priors from the final EMIRGE iteration. The data is available as a fasta file in the supplemental material and as GenBank SRA BioProject PRJNA483050.

### Phylogeny inference for 16S rRNA sequences

For Fig. 3, EMIRGE-reconstructed sequences (*n* = 5) were searched against SILVA SSU Reference NR sequences using the SINA 1.2.11 “search tool”^60^. For each Rifle sequence, sequences of neighbors with >95% sequence identity were collected. The reconstructed sequences and their close relatives were merged into a single file, sequence headers were annotated with the taxonomic labels, and aligned to SILVA seed alignment, and a maximum-likelihood phylogeny was inferred with RAxML^61^ (GTRGAMMA model of rate heterogeneity) using *Rhodopseudomonas palustris* HaA2 as an outgroup with 1000 bootstrapped trees to estimate node confidences (“raxmlHPC-SSE3 -f a -m GTRGAMMA -# 1000”). The resulting tree was visualized with Dendroscope^62^.

## Supplementary information

Supplementary Information

Dataset 1

Dataset 2

## Data Availability

Data that support the findings of this study are available within the paper, its supplementary information files, and data repositories cited therein, including NCBI GenBank Sequence Read Archive (SRA) (https://trace.ncbi.nlm.nih.gov/Traces/sra/sra.cgi) BioProject PRJNA483050. The relative relative abundances of each taxon are in Supplemental File 1 and the the reconstructed 16S rRNA gene sequence data for each taxon in FASTA format are in Supplemental File 2.
