# Supplementary material for: Paired RNA Radiocarbon and Sequencing Analyses Indicate the Importance of Autotrophy in a Shallow Alluvial Aquifer
Source: Sci Rep. 2019 Jul 17;9:10370. doi: 10.1038/s41598-019-46663-1 (PMC6637170; doi:10.1038/s41598-019-46663-1)
Supplement: Supplementary file 1 — Supplementary Information [file 41598_2019_46663_MOESM1_ESM.docx]

Supporting Information

for

Paired RNA Radiocarbon and Sequencing Analyses Indicate the Importance of Autotrophy in a Shallow Alluvial Aquifer

Submitted to

*Scientific Reports*

**Authors**: ^a^Brian J. Mailloux, ^a^Carol Kim, ^a^Tess Kichuk, ^a^Khue Nguyen, ^a^Chandler Precht, ^b^Shi Wang, ^b^Talia N. M. Jewell, ^b^Ulas Karaoz, ^b^Eoin L. Brodie, ^b^Kenneth H. Williams, ^b^Harry R. Beller, ^c^Bruce A. Buchholz

Affiliations:

^a^Environmental Science Department, Barnard College, NY, NY 10027

^b^ Earth and Environmental Sciences, Lawrence Berkeley National Laboratory, Berkeley, CA 94720

^c^Center for Accelerator Mass Spectrometry, Lawrence Livermore National Laboratory, Livermore, CA 94551-9900

Corresponding author:

Brian Mailloux

212-854-7956

bmaillou@barnard.edu

Running title: Ecological RNA analysis (radiocarbon and RNA-Seq)

Detailed RNA Extraction and Purification

The frozen filter was cut into ~1-inch rings with an electric saw with a sterile blade in a laminar flow hood. The plastic housing was removed and the four filter layers were separated, cut parallel to the pleats, and each was placed in a 50-mL tube. The extraction procedure focused on the outer shiny filter material, as this captured the majority of the bacteria. Twelve mL of a buffer with a final concentration of 2% CTAB, 1.4 M NaCl, 20 mM EDTA (pH 8), and 100 mM Tris-Cl (pH 8), with 64 mg lysozyme and 16 mg proteinase-K was added along with 1.7 g of 3-mm sterile beads. The mixture was vortexed on its side (tube held horizontally) for 30 sec and incubated for 30 min at 55°C. The buffer was decanted into a second 50-mL tube and 21 mL of 1x Tris-EDTA (TE) was added to the filter and vortexed horizontally for 30 sec. The TE was decanted into the second tube and any remaining TE/buffer in the filter was collected by squeezing with a 60-mL syringe. The combined buffer and TE was placed in 10-mL aliquots into 50-mL Phase Lock Gel (PLG) tubes (a.k.a. MaXtract High Density; Qiagen #129073, Qiagen, Hilden, Germany), which were made with a silicone lubricant (Mukhopadhyay and Roth, 1993). Equal amounts of 1:1 phenol (Fisher Scientific #bp1750I-400, Fisher Scientific, Pittsburgh, PA):chloroform-isoamyl alcohol (Fisher Scientific #AC327155000) (P:C) were added and shaken vigorously by hand until well mixed and centrifuged for 10 min at 1530 x *g* (3450 rpm) (Eppendorf 5804 centrifuge with rotor F-34-6-38, Eppendorf, Hamburg Germany). This was repeated once with 1:1 P:C and once with chloroform-isoamyl alcohol. The method was tested with both water saturated (pH 6.6) and tris saturated phenol (pH 7.9), the final procedure used tris saturated phenol (see results, Table 1). The remaining liquid was decanted to a new 50-mL tube, combined with an equal amount of isopropanol, and incubated on ice or in a freezer for 30 to 45 min. The RNA was pelleted for 20 min in 12 x 1.5-mL microcentrifuge tubes (USA Scientific, # 1615-5500, USA Scientific, Ocala, FL) at 21,130 x *g* (15,000 rpm) (Eppendorf 5424 centrifuge with rotor FA-45-24-11) in multiple steps until no liquid remained. The pellet was air-dried inverted for 60 min. The pellet in each tube was resuspended in 200 µL DNA-grade sterile water (Fisher #BP24701). RNA-grade sterile water was not used in order to avoid diethyl pyrocarbonate (DEPC). The 200 µL volumes from the tubes were combined and the RNA was precipitated a second time with one half the volume of 7.5M filter-sterilized ammonium acetate (ThermoFisher #bp326-500) and 3x the new total volume of 100% ethanol (Pharmco-Aaper #111000200, Pharmco-Aaper, Brookfield, CT). The pellets were washed with 1.5 mL of chilled 70% ethanol (Pharmco-Aaper #111000140) by vortexing for 10 to 20 sec and centrifuging at 21,130 x *g* (15,000 rpm) for 5 min. The wash was repeated once (two total washes) and the pellets were air-dried inverted for 60 min and then resuspended in 200 µL DNA-grade sterile water. The volumes were combined in a 15-mL tube and 5M filter-sterilized NaCl was added to make a final concentration of 0.2M NaCl and a second round of P:C treatment was performed. The second round of P:C was performed in either the 50 mL MaxXtract tubes or the 1.5 mL MaxXtract tubes (Qiagen #129046, Qiagen, Hilden, Germany) and this difference again was critical. The 1.5-mL MaxXtract tubes (Qiagen #129046, Qiagen, Hilden, Germany) used 250 µL of sample and were centrifuged at 21,130 x *g* (15,000 rpm) (Eppendorf 5424 centrifuge with rotor FA-45-24-11). The ethanol and isopropanol steps were repeated as above.

The resulting nucleic acids were a mixture of RNA and DNA. Purity and amounts were checked with a NanoDrop Spectrophotometer (ThermoFisher) and verified with a Qubit Fluorometer (ThermoFisher) using both RNA and DNA kits. Enzymes were not used to separate RNA and DNA, as they might have interfered with radiocarbon analysis. Instead, the RNA was precipitated with LiCl. All the samples from one filter/site were combined. The sample volume was reduced using a SpeedVac Concentrator (Savant ISS110, ThermoFisher). A 200-µL sample was combined with 300 µL DNA-grade sterile water and 250 µL of 7.5M LiCl (Fisher #ICN19401080) in a 1.5-ml microcentrifuge tube. The mixture was chilled at -20°C for 30 min and centrifuged at 21,130 x *g* (15,000 rpm) for 15 min. The pellet was decanted and washed twice with 70% ethanol, as described above. The pellet was resuspended in 200 µL DNA-grade sterile water and stored at -80°C until CsCl centrifugation and RNA-Seq or radiocarbon analysis.

Ultracentrifugation through a CsCl solution was also utilized for separating RNA from DNA and improving purity. The CsCl was baked at 450°C for 24 hr to remove impurities. 3.8 ml of sterile water was added to a 5-mL thin-wall centrifuge tube (#344057, Beckman Coulter, Brea, CA). One mL of 5.7M CsCl was added below the water using a syringe and 2 mL of sample was added to the top of the tube. This mixture was spun at 32,000 rpm for 19 hr at 25°C in a SW55Ti swinging bucket rotor. The pellet was resuspended in 0.5 mL of sterile water for 45 min at room temperature and precipitated with 1 mL of 100% ethanol with NaCl at -20°C for 45 min, followed by two 70% ethanol washes, as above.

RNA Controls

Different carbon sources were used to create end-member cases to the test the RNA extraction procedure by growing cells on that carbon source*.* The goal was to choose carbon sources with distinct radiocarbon signatures. Three carbon sources were chosen; acetate, lysogeny broth (LB), and dextrose.

Commercially available acetate is derived mostly from petroleum and thus possesses a highly negative radiocarbon signature (Δ^14^C) and a comparatively old C age. In practice, a sample with little to no radiocarbon has a Δ^14^C value approaching -1000‰ and contains carbon more than 50,000 years old. In contrast, LB and dextrose are derived from recent photosynthetically derived material and have a contemporary signature with a positive Δ^14^C value and a modern age (Table 1). Above-ground testing of nuclear weapons before the adoption of the Limited Test Ban Treaty of 1963 produced a bomb pulse of ^14^C in the atmosphere resulting in radiocarbon values with a fraction modern greater than 1 and a positive Δ^14^C value (Spalding et al., 2005). The use of the different growth media in the laboratory offered a controlled approach to validate the extraction and radiocarbon analysis procedure.

*E. coli* (strain ATCC 700891, ATCC, Manassas, VA) was grown with either acetate (Fisher #bp333-500) or dextrose (Fisher #D14-500) with M9 media (Sambrook, 2001) or lysogeny broth (LB) (Fisher #BP1426-500). Acetate is petroleum derived and is an older end-member, whereas LB contains yeast extract as a carbon source and provides a modern radiocarbon end-member along with dextrose. Cells were grown in multiple steps to remove previous carbon signatures. 100 µL of frozen cells were added to 50 mL of broth and incubated for 12 hr at 37°C. One mL of cells was removed and added to a second 50 mL aliquot of broth for 12 hr. Then, 1 mL of cells was removed and added to 1 L of broth for 12 hours. The cells were centrifuged in six 50-mL tubes at 3214 x *g* (5000 rpm) (Eppendorf 5804 centrifuge with rotor F-34-6-38) in batches until all cells were pelleted. The pellet was rinsed once with 5 mL of DNA-grade sterile water and then resuspended in 5 mL of DNA-grade sterile water and stored at -80°C. For RNA extractions, 1 mL of the resuspended pellet was added to the extraction buffer above instead of the filter.

RADIOCARBON DATA

Table S1. Well LQ107 Radiocarbon Results. DIC and DOC were analyzed at National Ocean Sciences Accelerator Mass Spectrometry (NOSAMS) (Woods Hole, MA). RNA was analyzed at Lawrence Livermore National Laboratory (LLNL) (Livermore, CA).

| Type | Accession # | Fraction Modern | ^14^C age (yrs) | Δ^14^C (‰) |
| --- | --- | --- | --- | --- |
| DIC | OS-131531 | 0.8488±0.0017 | 1,320±15 | -157.7± 1.7 |
| DOC | OS-131350 | 0.7749±0.0017 | 2,050±20 | -231.0± 1.7 |
| RNA | 169943 | 0.8129±0.0056 | 1660±60 | -193.4±5 |

Table S2. Radiocarbon Sediment data from Acid Washed Sediment. Data is from Janot et al., (2016). All data was analyzed at Beta Analytic (Miami, FL). The average Δ^14^C (‰) for the sediment carbon is -504.4±173.0 ‰.

| Sample Name | Accession # | Fraction Modern | ^14^C age (yrs) | Δ^14^C (‰) |
| --- | --- | --- | --- | --- |
| JD-01, 8-9' | 364449 | 0.3286±0.0016 | 8940±40 | -671.4±1.6 |
| JD-03, 9-9.5' | 364450 | 0.4132±0.0021 | 7100±40 | -586.8±2.1 |
| JD-03, 10-10.5' | 364451 | 0.3699±0.0018 | 7990±40 | -630.1±1.8 |
| JD-03, 11-11.5' | 366904 | 0.5522±0.0021 | 4770±30 | -447.8±2.1 |
| JD-03, 12-12.5' | 364453 | 0.3319±0.0017 | 8860±40 | -668.1±1.7 |
| LR-27, 9-12.5' | 364454 | 0.5536±0.0021 | 4750±30 | -446.4±2.1 |
| LR-27, 12.5-15' | 364455 | 0.4656±0.0023 | 6140±40 | -534.4±2.3 |
| LR-27, 15-17.5' | 364456 | 0.4486±0.0022 | 6440±40 | -551.4±2.2 |
| LR-27, 17.5-23.5' | 364457 | 0.2360±0.0015 | 11600±50 | -764.0±1.5 |
| LR-27, 23.5-25.5' | 364458 | 0.4215±0.0021 | 6940±40 | -578.5±2.1 |
| BH 2-1-13’ | 364459 | 0.3621±0.0018 | 8160±40 | -637.9±1.8 |
| BH 2-1-13 pyro-P extracted | 364460 | 0.3119±0.0016 | 9360 ±40 | -688.1±1.6 |
| JB-02; 13-13.5’ | 365918 | 0.7335±0.0027 | 2490±30 | -266.5±2.7 |
| JB-02; 13-13.5’ | 365919 | 0.5725±0.0021 | 4480±30 | -427.5±2.1 |
| JB-03; 12.5-13’ | 365920 | 0.5605±0.0021 | 4650±30 | -439.5±2.1 |
| JB-04; 9.5-10’ | 365921 | 0.3373±0.0021 | 8730±50 | -662.7±2.1 |
| JB-04; 13-13.5’ | 365922 | 0.8113±0.0030 | 1680±30 | -188.7±3.0 |
| JB-04; 14.75-15’ | 365923 | 0.8380±0.0031 | 1420±30 | -162.0±3.1 |
| JB-05; 9-10’ | 365924 | 0.5571±0.0021 | 4700±30 | -442.9±2.1 |
| JB-05; 13.5-13.75’ | 365925 | 0.7066±0.0026 | 2790±30 | -293.4±2.6 |

Table S3. Radiocarbon date from plant material (leaves and wood) collected from the shallow alluvial aquifer near Rifle, Colorado. Data is from Janot et al., (2016). Modern plant material was not included in the results following Janot et al., (2016). All data was analyzed at Beta Analytic (Miami, FL). The average Δ^14^C (‰) for the plant material is -55.1±61.8 ‰.

| Sample Name | Description | Accession # | Fraction Modern | ^14^C age (yrs) | Δ^14^C (‰) |
| --- | --- | --- | --- | --- | --- |
| FP-101; 17.5-20' | plant material | 364083 | 0.9131±0.0034 | 730±30 | -86.9±3.4 |
| CG-03; 18.25' | plant material | 364084 | 0.8113±0.0030 | 1680±30 | -188.7±3.0 |
| SY-05 just above Wasatch contact | plant material | 364085 | 0.9200±0.0034 | 670±30 | -80.0±3.4 |
| JB-02; 14-14.25’ | Wood | 357520 | 0.9864±0.0037 | 110±30 | -13.6±3.7 |
| JB-02; 13-13.5’ | plant material | 367917 | 0.9815±0.0037 | 150±30 | -18.5±3.7 |
| JB-04; 13-13.5’ | plant material | 367918 | 0.9852±0.0037 | 120±30 | -14.8±3.7 |
| JB-04; 14.75-15’ | plant material | 367919 | 0.9778±0.0037 | 180±30 | -22.2±3.7 |
| JB-05; 13.5-13.75’ | wood | 367920 | 0.9839±0.0037 | 130±30 | -16.1±3.7 |

REFERENCES

Janot, N., Lezama Pacheco, J.S., Pham, D.Q., O’Brien, T.M., Hausladen, D., Noël, V. et al. (2016) Physico-Chemical Heterogeneity of Organic-Rich Sediments in the Rifle Aquifer, CO: Impact on Uranium Biogeochemistry. *Environmental Science & Technology* **50**: 46-53.

Mukhopadhyay, T., and Roth, J.A. (1993) Silicone lubricant enhances recovery of nucleic acids after phenol-chloroform extraction. *Nucleic Acids Research* **21**: 781-782.

Sambrook, J.a.R., David (2001) *Molecular Cloning: A Laboratory Manual* Cold Spring Harbor, NY: Cold Spring Harbor Laboratory Press.

Spalding, K.L., Bhardwaj, R.D., Buchholz, B.A., Druid, H., and Frisen, J. (2005) Retrospective Birth Dating of Cells in Humans. *Cell* **122**: 133-143.
